# *Sc65*-Null Mice Provide Evidence for a Novel Endoplasmic Reticulum Complex Regulating Collagen Lysyl Hydroxylation

**DOI:** 10.1371/journal.pgen.1006002

**Published:** 2016-04-27

**Authors:** Melissa E. Heard, Roberta Besio, MaryAnn Weis, Jyoti Rai, David M. Hudson, Milena Dimori, Sarah M. Zimmerman, Jeffrey A. Kamykowski, William R. Hogue, Frances L. Swain, Marie S. Burdine, Samuel G. Mackintosh, Alan J. Tackett, Larry J. Suva, David R. Eyre, Roy Morello

**Affiliations:** 1 Department of Physiology & Biophysics, University of Arkansas for Medical Sciences, Little Rock, Arkansas, United States of America; 2 Department of Orthopaedics and Sports Medicine, University of Washington, Seattle, Washington, United States of America; 3 Department of Orthopaedic Surgery, Center for Orthopaedic Research, University of Arkansas for Medical Sciences, Little Rock, Arkansas, United States of America; 4 Department of Biochemistry & Molecular Biology, University of Arkansas for Medical Sciences, Little Rock, Arkansas, United States of America; 5 Division of Genetics, University of Arkansas for Medical Sciences, Little Rock, Arkansas, United States of America; Murdoch Childrens Research Institute, AUSTRALIA

## Abstract

Collagen is a major component of the extracellular matrix and its integrity is essential for connective tissue and organ function. The importance of proteins involved in intracellular collagen post-translational modification, folding and transport was recently highlighted from studies on recessive forms of osteogenesis imperfecta (OI). Here we describe the critical role of SC65 (Synaptonemal Complex 65, P3H4), a leprecan-family member, as part of an endoplasmic reticulum (ER) complex with prolyl 3-hydroxylase 3. This complex affects the activity of lysyl-hydroxylase 1 potentially through interactions with the enzyme and/or cyclophilin B. Loss of Sc65 in the mouse results in instability of this complex, altered collagen lysine hydroxylation and cross-linking leading to connective tissue defects that include low bone mass and skin fragility. This is the first indication of a prolyl-hydroxylase complex in the ER controlling lysyl-hydroxylase activity during collagen synthesis.

## Author Summary

Fibrillar collagens are major components of connective tissue extracellular matrix (ECM). Among them, type I collagen is the most abundant protein in the human body and a large constituent of bone, dermis, tendon and ligament ECMs; type I collagen is also present in the stroma of other organs including heart, lung and kidney where, when dysregulated, it significantly contributes to pathological fibrosis. Type I and other collagen molecules have triple-helical folding requirements and undergo numerous intracellular post-translational modifications in the endoplasmic reticulum (ER) and Golgi apparatus. We and others have shown that alterations/loss of specific collagen modifications can lead to severe congenital disease such as osteogenesis imperfecta (OI). Here, using a multidisciplinary approach, we describe functional studies of the SC65 protein (Synaptonemal Complex 65 or P3H4), a poorly characterized member of the Leprecan gene family of proteins. We provide evidence that SC65 is a critical component of an ER complex with prolyl 3-hydroxylase 3 (P3H3), lysyl-hydroxylase 1 (LH1), and potentially cyclophilin B (CYPB). Loss of Sc65 in the mouse results in instability of this complex, site-specific reduction in collagen lysine hydroxylation and connective tissue defects including osteopenia and skin fragility.

## Introduction

Fibrillar collagens are abundant components of connective tissue extracellular matrix (ECM) [1]. They are formed by three polypeptides (termed α chains) each characterized by the presence of a long uninterrupted Gly-X-Y sequence repeat which folds into a characteristic triple-helical structure [1,2]. Among them, type I collagen (α1(I)_2_α2(I)) is the most abundant protein in the human body and the major constituent of bone, dermis, tendon and ligament ECMs. It is also expressed in the stroma of other organs including heart, lung and kidney where, when dysregulated, it can significantly contribute to pathological fibrosis [3]. Type I and other collagens undergo many intracellular post-translational modifications in the endoplasmic reticulum (ER) and Golgi apparatus [4]. For this reason, type I collagen has been considered a ‘prototypical model’ for mechanistic studies of enzymes that modify specific residues and chaperone proteins that facilitate folding of newly synthesized polypeptides in the secretory pathway. Intracellular modifications of collagens are critical for the structural integrity of the ECM (e.g. specific lysine residues in α1(I) and α2(I) can be hydroxylated and glycosylated and go on to form intermolecular cross-links in the ECM [5]). The extent of these modifications can also be tissue-specific and thus provide different biochemical properties to the same primary amino acid sequence of collagen [6]. With the recent identification of mutations in multiple genes encoding proteins involved in collagen post-translational modification and folding [7,8,9,10,11,12], the importance of these processes in human disease has become evident.

In the ER, specific proline and lysine residues of newly translated procollagen chains are modified by prolyl- and lysyl-hydroxylases, respectively. These enzymes share a highly conserved catalytic 2-oxoglutarate, ascorbate- and Fe(II)-dependent dioxygenase domain [13]. Collagen prolyl 4-hydroxylases (P4Hs) modify proline residues in the Y position of the Gly-X-Y repeat into 4-hydroxyproline (4Hyp). This frequent modification confers thermal stability to the folded triple-helical structure [14]. Interestingly, P4Hs form a tetrameric complex (α2β2) where the alpha (α) subunit is one of three prolyl 4-hydroxylase genetic isoforms and the beta (β) subunit is protein disulfide isomerase (PDI) [15]. Modification of proline residues into 3-hydroxyproline (3Hyp) by collagen prolyl 3-hydroxylases occurs far less frequently and always on proline residues in the X position of a Gly-X-4Hyp repeat (most completely on Pro^986^ of α1(I)) [16]. We previously demonstrated that CRTAP, a non-enzymic member of the Leprecan family of proteins which includes Synaptonemal Complex 65 (SC65) and the prolyl 3-hydroxylases (P3H1, P3H2 and P3H3), is an essential third subunit of a complex with prolyl 3-hydroxylase 1(aka LEPRECAN) and cyclophilin B (CYPB) in the ER, forming the so-called collagen prolyl 3-hydroxylation complex [17]. Loss of CRTAP in mice resulted in loss of the complex, lack of Pro^986^ hydroxylation, defective collagen fibrillogenesis and severe osteopenia. Indeed, human mutations in *CRTAP* cause recessive osteogenesis imperfecta (OI) [17]. This discovery then led to the identification of mutations in additional genes encoding ER-resident proteins that included P3H1, CYPB, FKBP65, HSP47 and OASIS, all causing recessive forms of OI [7,8,9,10,11,12].

Collagen lysyl-hydroxylases (LH1, LH2 and LH3) usually modify the side chain of lysine residues in the Y position of the Gly-X-Y repeat into hydroxylysine (Hyl) [5]. Certain triple-helical domain hydroxylysines can be o-glycosylated in the Golgi. Lysine and Hyl in collagen telopeptide domains are substrates for lysyl oxidase and the resulting aldehydes form cross-links in fibrils with helical domain lysine, Hyl or glycosylated Hyl side-chains [5,18]. Importantly, collagen lysyl-hydroxylases have not yet been formally demonstrated in a specific protein complex. In this study we describe the functional characterization of SC65, a protein that by primary sequence is closely related to CRTAP with as yet no known function. Using a multi-disciplinary approach combining mouse genetics, cell biology, microscopy, proteomics, and biochemistry, we demonstrate that SC65 is a critical component of an ER complex with prolyl 3-hydroxylase 3 (P3H3), lysyl-hydroxylase 1 (LH1), and potentially CYPB. The loss of SC65 in a mouse genetic model results in instability of this ER complex, leading to severely depleted lysine hydroxylation at helical domain cross-linking sites, disordered fibrils and significant defects in connective tissues that include osteopenia and skin fragility.

## Results

### Generation of a *Sc65KO* mouse model

**Fig 1:**
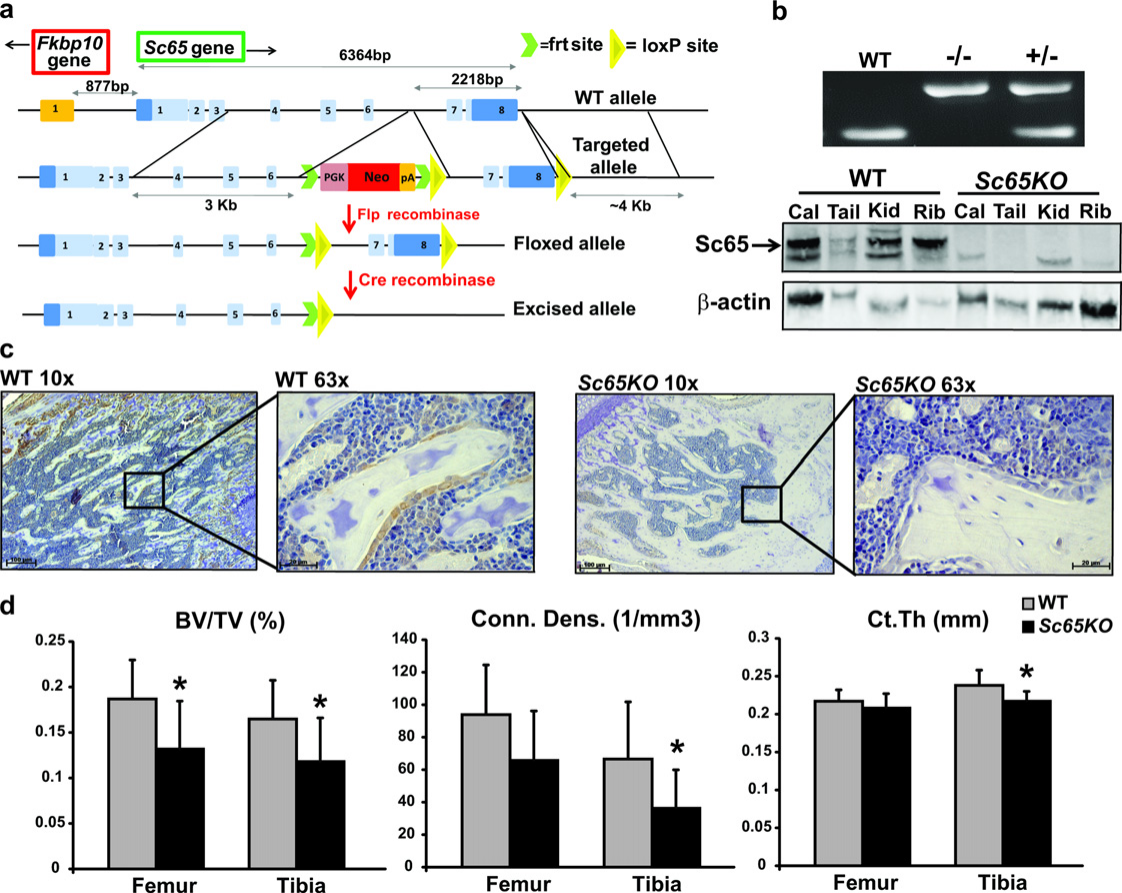
*Sc65KO* mouse generation and confirmation of bone loss phenotype. a) Strategy for the creation of the *Sc65-null* allele. The schematic diagram shows the *Sc65* wild-type, targeted, floxed and excised allele. *Sc65* coding regions are in light blue while non-coding regions are in dark blue. Also note the proximity to the *Fkbp10* gene which is transcribed in the opposite orientation. b) PCR genotyping of *Sc65KO* mice (upper panel) and Western blot confirmation of SC65 protein (arrow) loss in multiple *Sc65KO* tissues from 3 day-old mice compared to WT controls (lower panel—Cal = calvaria, Kid = Kidney). c) Immunohistochemistry detection of SC65 in adult femur section from a WT mouse showing specific intracellular staining in bone forming cells (osteoblasts) aligned on the surface of a bone trabecula. *Sc65* expression is lost in a similar section from a *Sc65KO* mouse. Scale bars = 100μM (10x) or 20μM (63x). d) MicroCT analysis of long bones from 6 month-old WT and *Sc65KO* male mice (n = 9). Both femurs and tibias from *Sc65KO* mice exhibited decreased trabecular bone volume/tissue volume (BV/TV), connectivity density (Conn.D) and cortical thickness (Ct.Th) compared to WT controls (*p<0.05).

We have shown that SC65, similar to other Leprecan members, is an ER-associated protein that is expressed in multiple tissues and highly enriched in bone, cartilage, skin and kidney [19]. Using an *Sc65* loss-of-function mouse model a low bone mass phenotype was observed [19]. However, no effect on prolyl 3-hydroxylation was detected and the molecular function of SC65 remained unknown. The original murine model (involving a gene-trap insertion) contained a neomycin-resistance cassette driven by its own promoter which, in theory, could have impacted the expression of nearby genes. For instance the *Fkbp10* gene lies less than 900bp from *Sc65* exon 1, is transcribed in the opposite direction and likely shares common regulatory elements. Importantly mutations in *FKBP10* have been associated with recessive OI [10]. Therefore to eliminate this possibility and confirm the bone-related function for SC65, we report here the generation of a new *Sc65-null* allele. Rather than targeting the 5’ end of the gene, with the potential risk of interfering with *Fkbp10* expression, LoxP sites were introduced flanking the last two exons of *Sc65* (Fig 1A).

Heterozygous mice carrying the new floxed allele were mated first with mice expressing Flp-recombinase to eliminate the neomycin selection cassette, and then with mice expressing Cre-recombinase driven by an early ubiquitous promoter to generate a global KO (Fig 1A). Mice carrying the *Sc65* Cre-excised allele (herein referred to as *Sc65KO*) were bred to homozygosity and western blot analysis of extracts from 3 day-old mouse tissues confirmed complete loss of SC65 protein (Fig 1B), indicating that the Cre-excised allele is a null-allele. This result was further supported by immunohistochemistry using an SC65 polyclonal antibody which did not stain osteoblasts on sections of *Sc65KO* compared to WT long bone (Fig 1C). Moreover, real-time PCR analyses confirmed rapid degradation of the SC65 truncated transcripts by NMD and additional western blot showed lack of potential truncated SC65 protein products in *Sc65-KO* primary skin fibroblasts (S1 Fig). These *ex vivo* data confirmed the expression of SC65 in bone forming osteoblasts and the validity of the new mouse model. *Sc65KO* mice were born at the expected Mendelian ratio and did not show any macroscopic differences compared to their WT littermates. To confirm the previously described osteopenic phenotype [19], micro-CT analysis of both femur and tibia of 6 month-old male mice (n = 9) was performed. A statistically significant decrease in trabecular bone volume/tissue volume (BV/TV) was detected in *Sc65KO* compared to WT long bones (p<0.05) (Fig 1D). Consistent with this, the connectivity density (Conn.D), which quantifies the connected structures within the trabecular bone network, was also significantly reduced in *Sc65KO* long bones. Moreover, the bone cortical thickness at the diaphysis differed significantly between the two genotypes (Fig 1D). Additional bone micro-CT measurements were acquired and were consistent with those reported for the previous mouse model of *Sc65* inactivation [19], confirming a specific role for SC65 in the accrual/maintenance of normal bone mass.

### SC65 protein interactions profiled by proteomic mass spectrometry

To begin to investigate the function of SC65, we adopted a co-immunoprecipitation approach to identify SC65 candidate protein interactors. Murine 714 cells, a transformed mouse fibroblast cell line derived from BALB/3T3 [20], were transiently transfected with a SC65-Flag expression construct or an empty vector control and the lysates immune-precipitated (IP) using a Flag antibody and separated by gel electrophoresis (S2A Fig). IP fractions from both samples were analyzed and quantified by mass-spectrometric analysis. A total of 253 proteins, enriched at least 1.5 fold in the IP fraction of cells transfected with the SC65-Flag construct compared to the empty control vector, were identified as candidate SC65 interacting partners (S2B Fig). Among these proteins were several alpha-chains from different types of fibrillar collagen, including type V, I and XI (Table 1) strongly implicating SC65 in collagen processing and/or trafficking.

**Table 1:** Candidate fibrillar collagen interactors of SC65. Fold change indicates enrichment relative to IP from cells transfected with empty vector.

Collagen chains	Fold Change
α2(V)	47.7
α1(V)	40.4
α1(I)	5.7
α1(XI)	3.7
α2(XI)	2.4
α2(I)	1.7

### SDS-PAGE of collagen extracted from bone and skin

**Fig 2:**
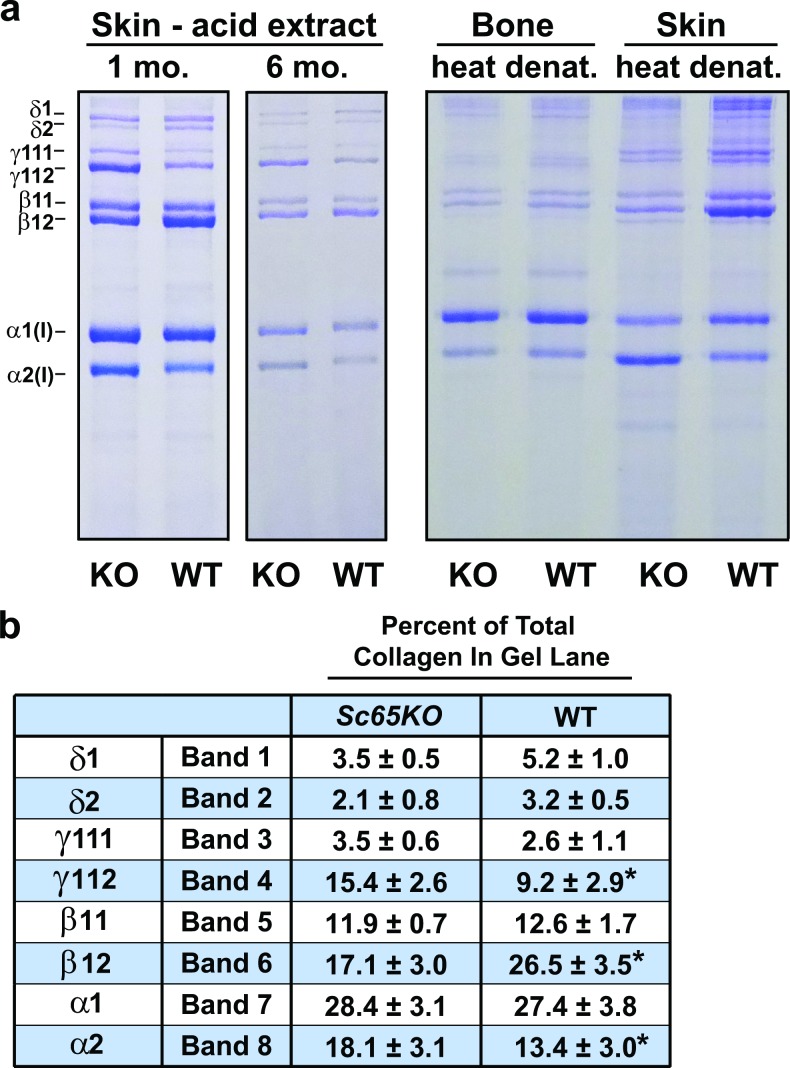
Increased electrophoretic mobility and altered cross-linking of type I collagen from *Sc65-null* skin. a) SDS-6%PAGE of type I collagen extracted from skin and decalcified bone of *Sc65KO* and WT mice shows increased mobility of α-chains and reduced ratio of cross-linked β to γ components in the *Sc65KO* skin extracts. An acetic acid extract from skin of the original Sc65-null mouse^19^ (1 mo.) created by gene-trap insertion is compared with that from the new *Sc65KO* (6 mo.) and their respective WT controls. Total heat denatured extracts of skin and bone collagens from new *Sc65KO* mice are shown on the right for comparison. Bone collagen from *Sc65KO* mice does not show the differences from WT in β/γ intensities evident for skin collagen. The strong (lower) γ band in SC65 skin extracts was identified as γ _112._ Both original and new *Sc65KO* mice showed the same altered pattern of chain intensities from WT most pronounced in the acetic acid extracts of skin with an apparent increase in γ_112_ at the expense of β_12._ b) Densitometric analysis of collagen bands on SDS-PAGE. Densitometry was performed on bands 1–8 (counted from top to bottom) of acetic acid extracts from 1mo and 6mo skin samples of both original and new *Sc65KO* mice using NIH imageJ software. Values are means ± SD, n = 6; *p<0.01.

A role for SC65 in collagen post-translational modification was first indicated experimentally by SDS-PAGE analysis of extracts of skin collagen which consistently showed reproducible differences between *Sc65KO* and WT mice (Fig 2). The visual differences between null and WT SDS-PAGE banding patterns were reproducible across multiple extracts and gels.

Densitometry to quantify gel bands showed a significant decrease in the β_12_ dimer and an increase in γ_112_ (Fig 2B). The ratio of α1(I) to α2(I) also differed with α2(I) being more prominent relative to α1(I) in mutant skin extracts. In addition, a modest but consistent increased mobility was evident for both α1(I) and α2(I) chains from the *Sc65KO* dermis based on multiple SDS-PAGE gels using tissue from 1 month- and 6 month-old mice. The latter observation suggested decreased lysine hydroxylation and/or glycosylation in the triple-helical domain. Heat denatured extracts of decalcified bone collagen (required to see sufficient soluble collagen on SDS-PAGE) showed no marked electrophoretic differences in cross-linked chain properties and less effect on mobility of α-chains from *Sc65KO* versus WT bone.

### Lack of SC65 has no effects on collagen prolyl 3-hydroxylation

Collagen α1(I) and α2(I) chains excised from SDS-PAGE gels (Fig 2) were subjected to in-gel trypsin digestion and tandem mass spectral analysis to quantify collagen post-translational modifications. Given the role of Crtap in prolyl 3-hydroxylation, known sites of 3-hydroxyproline (3Hyp) were initially targeted. As reported for the original gene-trap *Sc65KO* mice [19] no known prolyl 3-hydroxylation sites showed any significant suppression in level of 3Hyp in the *Sc65KO* mice (–see S1 Table for results for type I collagen from skin and bone).

### SC65 loss impairs collagen triple-helical lysine hydroxylation

**Fig 3:**
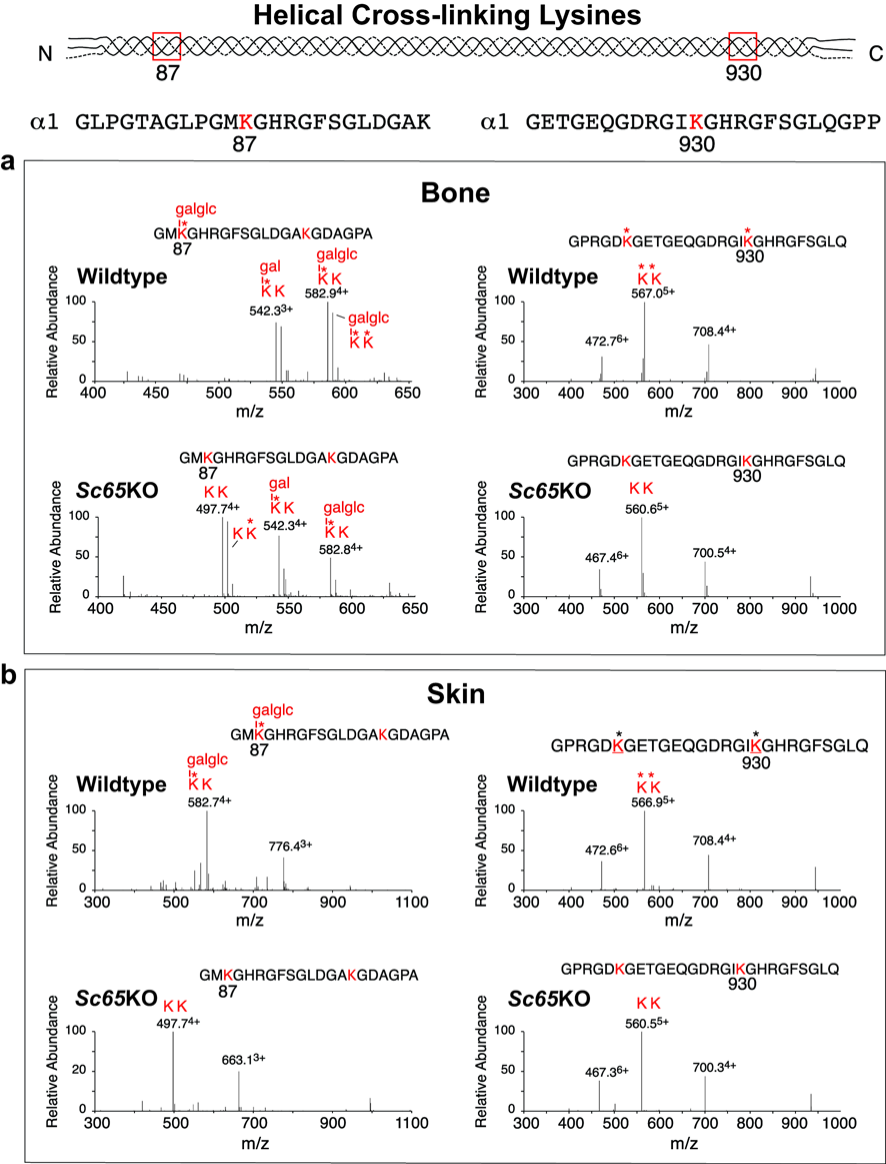
Undermodification of lysine residues at cross-linking sites in Sc65-null skin and bone collagens revealed by tandem mass spectrometry. Peptides from the two helical domain cross-linking sites in collagen α1(I) prepared by bacterial collagenase digestion of decalcified bone (a) and skin (b) were identified by LC-MS. Their post-translational profiles are compared between *Sc65KO* and wild type. a) Spectra from the two helical sites (Lys-87 and Lys-930) in bone show that Lys-87 is essentially all glcgalHyl plus galHyl in wild type and about 40% is lysine plus hydroxylysine in mutant; Lys-930 is all hydroxylysine in wild type and all lysine in mutant. b) Spectra from the two helical sites (Lys-87 and Lys-930) in skin show that Lys-87 is all glcgalHyl in wild type and all lysine plus hydroxylysine in mutant; Lys-930 is all hydroxylysine in wild type and all lysine in mutant. (glcgal = glucosyl-galactosyl; gal = galactosyl; * = hydroxyl group).

Based on the increased collagen α1(I) and α2(I) chain mobility and disturbed cross-linked chain ratios, tandem mass spectral analyses were also directed at sites of lysine hydroxylation, particularly those at cross-linking sites. In-gel trypsin digested collagen type I chains initially revealed marked under-hydroxylation at helical cross-linking sites K87 and K930/933 for *Sc65KO* compared to WT skin. These residues are preferred substrates for LH1 activity [21]. To confirm this more quantitatively for whole tissue collagen and also target collagen telopeptide lysines, bacterial collagenase digests of decalcified bone and skin were analyzed by tandem mass spectrometry. The results are summarized in Fig 3 and confirm a marked under hydroxylation of K87 and K930 in α1(I) of bone (a) and skin (b).

At K87, which normally is also glycosylated (mostly glcgalHyl in skin, galHyl in bone), the under-modification is more complete in skin than bone perhaps explaining the more obvious α-chain increased mobility for skin collagen (Fig 2). The α2(I) chain also showed under-hydroxylation at its equivalent two crosslinking sites K87 and K933. Neither N-telopeptide nor C-telopeptide hydroxylysine content, which requires LH2 activity [22], was found to differ between *Sc65KO* and WT bone or skin collagen α1(I) or α2(I) chains.

### Pyridinoline (HP and LP) crosslink analysis

Based on the observed under hydroxylation of cross-linking lysines in *Sc65KO* mouse bone and skin collagen, bone collagen was acid hydrolyzed and analyzed by HPLC to quantify pyridinoline cross-links. The results showed a reversed ratio of hydroxylysylpyridinoline (HP) to lysylpyridinoline (LP) of 0.3/1 from *Sc65KO* bone compared with 5/1 from WT bone. This is consistent with the mass spectral data on linear peptides showing severe under-hydroxylation of the helical-domain lysines that control whether HP or LP cross-links are formed. The total content of HP+LP pyridinoline cross-links was in the normal range for mouse bone from both genotypes, consistent with the mass spectral findings that linear N- and C-telopeptide lysine hydroxylation was unaffected.

### Targeted identification of SC65 interacting proteins defines a new ER complex

The severe reduction of collagen triple-helical lysine hydroxylation identified by mass-spectrometry indicated that LH1 function is dependent on the presence of SC65 protein. Importantly, in our previous mass-spectrometry screening of potential SC65 interactors, LH1 was 4.9 fold enriched in the IP fraction derived from cells transfected with the SC65-Flag construct compared to controls, suggesting that SC65 and LH1 may cooperate in a protein complex.

**Fig 4:**
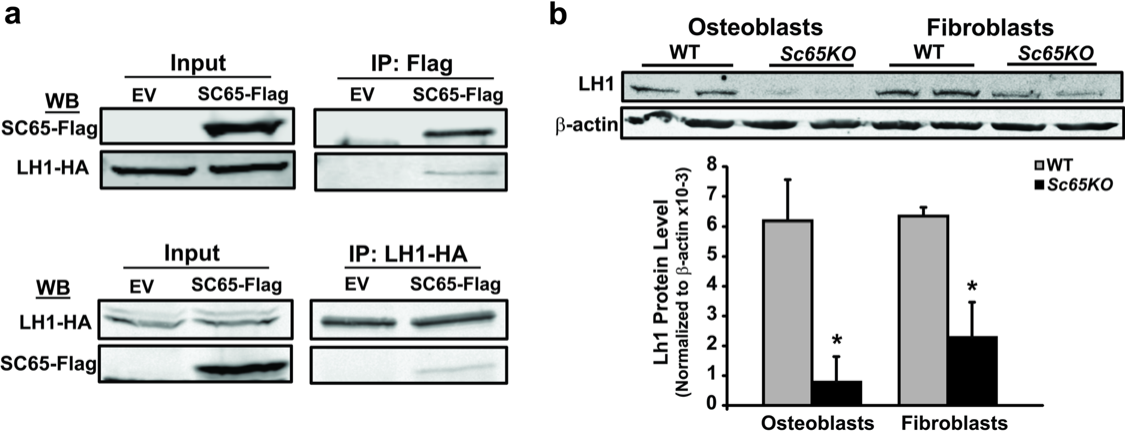
SC65 directly interacts with lysyl-hydroxylase 1 (LH1). a) Murine 714 mouse embryonic fibroblasts stably expressing SC65-Flag or an empty vector (EV) control were transiently transfected with a HA-tagged Lh1 and lysed 48 hours after transfection. Immuno-precipitation (IP) experiments were conducted using a Flag antibody (upper panel) or a HA antibody (lower panel). 10% of total inputs and immuno-precipitates were separated on a 10% SDS-PAGE gel, blotted and probed with antibodies against FLAG and HA. The reciprocal interaction of SC65-Flag with LH1-HA is confirmed in both experiments. b) Western blot of primary calvarial osteoblast and skin fibroblast lysates from WT and *Sc65KO* 3 day-old mice (N = 2) showing significantly decreased levels of LH1 protein in *Sc65KO* samples. Densitometric quantification of LH1 protein normalized to β-actin from the western blot shown above (*p<0.05; error bars represent SD). All experiments were performed at least 3 times.

To confirm this specific interaction 714 cell lines stably expressing SC65-Flag or an empty vector (EV) control were developed and transiently transfected with an HA-tagged LH1 expression plasmid. Direct co-immunoprecipitation assays utilizing either a Flag or HA antibody were then performed, followed by Western blotting with the HA or the Flag antibody or both. These assays confirmed a reciprocal and direct interaction between SC65 and LH1 (Fig 4A).

Moreover, Western blots of primary calvarial osteoblast and skin fibroblast lysates from *Sc65KO* mice consistently demonstrated a significant decrease in LH1 protein compared to WT controls (Fig 4B). This important result indicated that loss of SC65 results in partial loss of LH1 protein.

**Fig 5:**
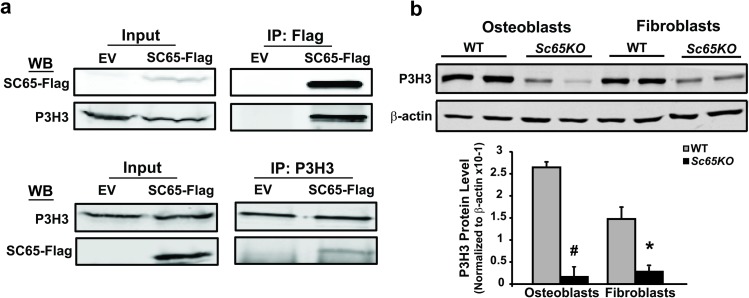
SC65 directly interacts with prolyl 3-hydroxylase 3 (P3H3). a) Lysates of 714 mouse embryonic fibroblasts stably expressing SC65-Flag or EV control were used for IP experiments utilizing a Flag antibody (upper panel) or a P3H3 antibody (lower panel). 10% of total inputs and immuno-precipitates were separated on a 10% SDS-PAGE gel, blotted and probed with antibodies against FLAG and P3H3. The reciprocal interaction of SC65-Flag with P3H3 is confirmed in both experiments. b) Western blot of primary calvarial osteoblast and skin fibroblast lysates from WT and *Sc65KO* 3 day-old mice (N = 2) showing significantly decreased levels of P3H3 protein in *Sc65KO* samples. Densitometric quantification of P3H3 protein normalized to β-actin from the western blot shown above (#p<0.01; *p<0.05; error bars represent SD). All experiments were performed at least 3 times.

Considering the close homology between CRTAP and SC65 and the knowledge that CRTAP is required to stabilize the P3H1/CRTAP/CYPB protein complex, we hypothesized that LH1 is associated with a similar complex. Significantly, mice with loss of function of *Leprel2* (encoding P3H3) have the same loss of tissue type I collagen lysine-hydroxylation as that observed in the *Sc65KO* mice when analyzed by tandem mass spectrometry (Hudson et al, in preparation). This suggested that P3H3 was also part of a SC65/LH1 complex. A direct interaction between SC65 and P3H3 was confirmed by a similar co-IP procedure to that described above, using 714 cells stably expressing SC65-Flag or an EV control but using primary antibodies against P3H3 or the Flag tag for the pull down (Fig 5A).

**Fig 6:**
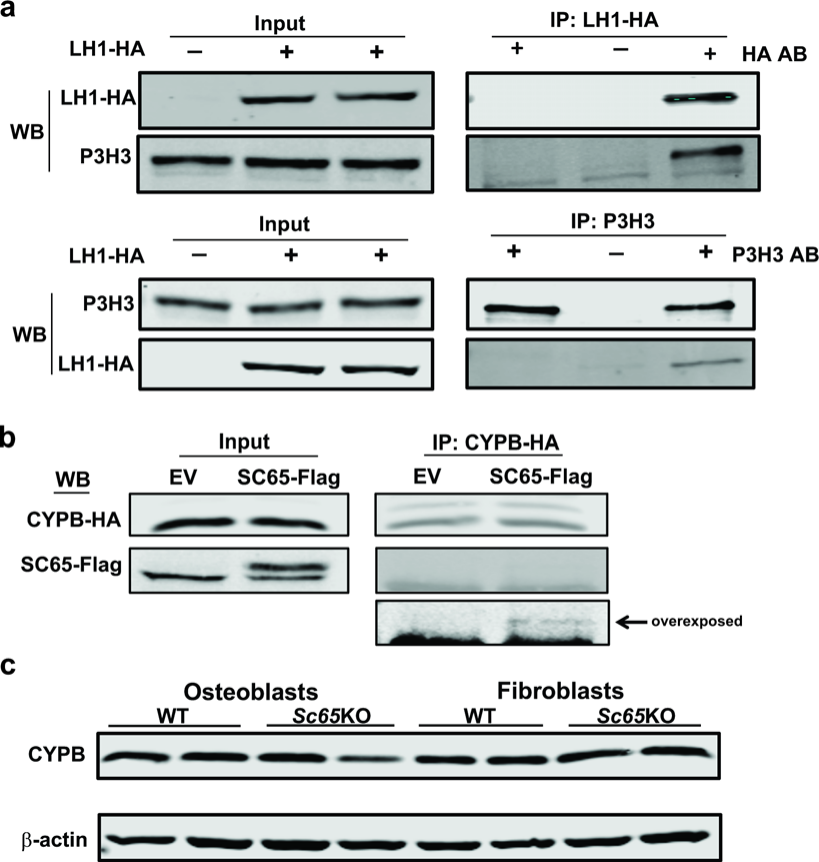
Characterization of a new SC65/LH1/P3H3 complex in the ER. a) Lysates of 714 mouse embryonic fibroblasts that were transiently transfected with an HA-tagged LH1 expression construct were immuno-precipitated with a HA antibody (upper panel) or a P3H3 antibody (lower panel). 10% of total inputs and immuno-precipitates were separated on a 8% SDS-PAGE gel, blotted and probed with antibodies against HA and P3H3. Negative controls included non-transfected 714 cells incubated with the HA antibody (for non-specific binding of HA antibody, left lanes) and LH1-HA transfected cells incubated with no antibody (for non-specific proteins binding to beads, middle lanes). In both experiments, LH1-HA and P3H3 were found to interact (right lanes). b) Lysates of 714 mouse embryonic fibroblasts stably expressing SC65-Flag or EV control and transiently transfected with a HA-tagged CYPB were used for IP utilizing an HA antibody. 10% of total input and immuno-precipitates were separated on a 12% SDS-PAGE gel, blotted and probed with antibodies against Flag and HA. The blot detecting SC65-Flag following IP with the HA antibody is shown over-exposed. c) Western blot of primary calvarial osteoblast and skin fibroblast lysates from WT and *Sc65KO* 3 day-old mice (N = 2) showing similar content of CYPB protein in *Sc65KO* and WT samples. All experiments were performed at least 3 times.

Also in this case, Western blot analysis showed that lack of SC65 in mouse primary calvarial osteoblasts and skin fibroblasts results in severe loss of P3H3 protein compared to WT control cells (Fig 5B). We then transiently transfected 714 cells with HA-tagged LH1 and successfully co-precipitated P3H3 or LH1, using either a HA-tag or P3H3 antibody, respectively (Fig 6A).

This strongly suggested that SC65/LH1/P3H3 are interlinked within a protein complex in the ER. To assess whether CYPB could also be part of such complex, as in the P3H1/CRTAP/CYPB complex, we transfected 714 cells with HA-tagged CYPB and successfully co-precipitated SC65-Flag using the HA antibody (Fig 6B). Notably, initial evidence for a potential interaction between LH1 and Cyclophilin B was recently provided [23,24]. To further pursue the nature of the suspected SC65/P3H3/LH1/CYPB complex we performed preparative, high resolution size exclusion chromatography. 714 cells stably transfected with SC65-Flag were used in multiple large IP experiments using the monoclonal antibody against Flag. To maintain its integrity the protein complex pulled down in the IP was eluted from the magnetic beads by competitive binding with an excess of Flag peptide and the eluted suspension was then loaded and run on a Superdex 200 column. Collected fractions were concentrated, run on SDS-PAGE and blotted with antibodies against members of the proposed complex (S3 Fig). SC65 (detected by the Flag antibody) and P3H3 protein co-eluted in fractions 23–26 (equivalent to an estimated MW of about 250kDa). LH1 protein peaked in fractions 28–30 with trace amounts in fractions 25–27. Taken together these findings demonstrate the existence of an ER protein complex that includes SC65/P3H3 and LH1 but no evidence so far that CYPB is included. We have also observed that lack of SC65 significantly reduces the content of LH1 and P3H3 in primary osteoblasts and fibroblasts, but not of CYPB (Fig 6C).

### Defective collagen cross-links in *Sc65KO* mice cause skin fragility and altered collagen fibrillogenesis

**Fig 7:**
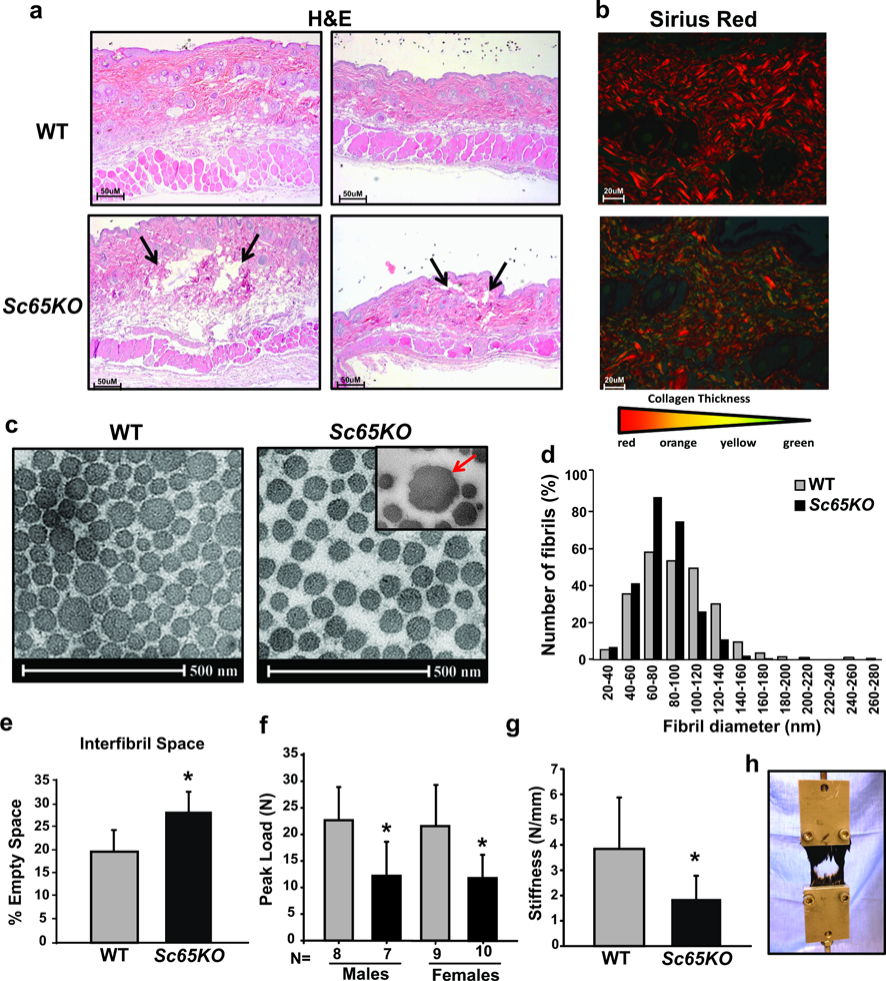
Loss of Sc65 results in dermal tears, abnormal collagen fibrils and skin fragility. a) H&E stained sections of WT and *Sc65KO* skin. Note the decreased density of collagen, the frayed dermis indicated by arrows and the reduced thickness of the muscle layer in the *Sc65-null* samples. b) Serial skin sections were stained with Sirius red. *Sc65-null* skin exhibits fewer large collagen fibers (red staining) and greater number of smaller collagen fibers stained in green compared to WT counterparts. c) Electron micrographs of 7 month-old mouse skin biopsy from WT and *Sc65KO* mice. Collagen fibrils, shown in cross-section, from *Sc65-null* skin tended to be smaller and have a decreased range of fibril diameter compared to WT fibrils. Loss of Sc65 also resulted in the presence of collagen fibrils with irregular profile and several large “cauliflower-like” fibrils (red arrow) which indicate abnormal fibrillogenesis (scale bar represents 500nm). d) Distribution of collagen fibril diameter in WT and *Sc65KO* mouse skin as measured from electron microscopy images. Measurements were collected from three different mice/genotype and >200 fibril/mouse. e) Skin EMs from *Sc65KO* mice also exhibited significantly more empty space among collagen fibrils compared to WT mice indicating a less densely packed collagen (*p = 0.01). Five electron micrograph images of non-overlapping areas were quantified from each mouse. f-h) Skin samples from WT and *Sc65KO* mice were subjected to a biomechanical skin loading test to measure tensile strength. Skin that lacks SC65 expression ruptured at a significantly lower peak load compared to WT skin indicating significant skin fragility (*p<0.01).

Because collagen cross-links are essential for the integrity of the dermis as well as the tensile properties of skin [25,26], we harvested biopsies of dorsal skin from *Sc65KO* and WT mice for light microscopy, visualization of collagen fibrils under polarized light and electron microscopy and biomechanical testing. Light microscopy of skin sections stained with hematoxylin & eosin showed decreased density of collagen fibers in the dermis, thinning of the muscle layer below the hypodermis and frequent presence of tears in the *Sc65KO* compared to WT sections (Fig 7A).

Serial skin sections were also labeled with Sirius Red, a collagen specific stain, and visualized under polarized light. The birefringent pattern of collagen in skin from *Sc65KO* mice appeared different compared to WT controls, and suggested a decreased size of collagen fibers that tended to be more green than orange [27] (Fig 7B).

Immunohistochemical analysis using the anti-SC65 antibody showed SC65 expression in WT dermal fibroblasts and lack of specific signal in skin sections from *Sc65KO* mice (S3 Fig). Ultrastructural analysis of dermal collagen from skin of 7 month-old *Sc65KO* mice revealed several differences compared to WT controls. Mutant dermal collagen fibrils were packed less orderly than age-matched wild type and their diameters were more uniform than WT fibrils (Fig 7C and 7D). Thus, 91.7% of mutant fibrils ranged from 40 to 120 nm in diameter compared with 78.7% of WT fibrils. The cross-sectional profile of collagen fibrils from *Sc65KO* skin was more disordered than WT with occasional thick and abnormally shaped fibrils with irregular contours, resembling ‘cauliflower-shaped’ fibrils [28] (Fig 7C inset). These fibrils are considered a hallmark of disturbed collagen fibrillogenesis, and are frequently observed in dermis from Ehlers-Danlos syndrome patients [29]. Furthermore, the quantified space between fibrils in *Sc65KO* skin was greater than in WT controls (Fig 7E). These findings suggest defects in the maturation and the lateral fusional growth of fibrils.

Finally, it is generally accepted that skin tensile strength correlates directly with the overall organization, content, and physical properties of the collagen fibril network [30]. Therefore, to obtain an objective measurement of skin fragility, we determined the tensile strength in portions of dorsal skin from wild-type and mutant mice. In load-to-failure biomechanical testing, skin from *Sc65KO* animals ruptured prematurely at a peak load of approximately 12N compared to WT skin that ruptured at approximately 23N (p<0.01)(Fig 7F and 7H). The tensile failure loads were reduced equally in both mutant male and female skin samples. The skin stiffness was also significantly reduced in *Sc65KO* compared to WT mice (Fig 7G). The finding of decreased tensile strength and stiffness directly support the histological observation of skin fragility and decreased collagen density.

## Discussion

The results presented demonstrate that SC65 is required for the normal post-translational modifications of collagen chains in the ER. This function is clearly distinct from, but related to, that of CRTAP, a highly homologous protein (~55% identical in amino acid sequence) that we have shown is essential for prolyl 3-hydroxylation^17^. Similar to CRTAP, SC65 appears necessary in another protein complex involved in the post-translational modifications of fibrillar collagen. While CRTAP binds to and forms a stable complex with P3H1 [17], SC65 forms a stable complex with P3H3. The formation of this complex is strongly supported by size-exclusion chromatography where SC65 and P3H3 eluted in the same fractions. However, what is unexpected is the specific effect of SC65 on collagen lysyl hydroxylation rather than on prolyl 3-hydroxylation. Our findings indicate that the SC65/P3H3 complex also involves LH1 and that the loss of SC65 results in at least partial loss of LH1 protein and activity. Interestingly, while some degree of overlap exists in chromatography fractions containing SC65, P3H3 and LH1, suggesting they can exist within a single complex, the later elution of LH1 also suggests a less stable interaction with the SC65/P3H3 core and dissociation from the complex during elution with Flag peptide and/or in less than ideal conditions such as those present in the column. It is also important to note that LH1 is not present in fractions that contain only SC65 or only P3H3, therefore it is unlikely that LH1 forms a complex with either of these proteins alone but rather with both of them together. While the precise stoichiometry of the complex is not yet defined, a putative trimeric complex containing SC65/P3H3/LH1 would have an estimated MW>200 kDa. These findings provide the first description of a prolyl-hydroxylase complex in the ER that affects lysyl-hydroxylation of collagen. However, while loss of SC65 causes under-hydroxylation of helical lysine residues, we have not been able to detect loss of prolyl 3-hydroxylation at any known site in collagen I from bone or skin from either *Sc65KO* (Suppl. Table 1) or *P3h3-null* mice (Hudson et. al., in preparation). Either the enzyme activity of P3H3 is redundant (e.g. compensated by P3H2) or lost during evolution but complex formation and collagen chaperone function are retained.

Although we show that SC65 is capable of interacting with Cyclophilin B *in vitro* (Fig 6), as might be expected given its homology to CRTAP, the results of gel filtration chromatography (S3 Fig) imply that in the ER this is likely a weak or transient interaction. It is also notable that mutations in PPIB (encodes CYPB) depending on their location can cause not only recessive OI [8,9] but also a severe form of skin fragility and blistering in American quarter horses, known as HERDA [23,31]. Therefore, mutations affecting different regions of CYPB can have different tissue effects, perhaps as a result of which ER complex function is compromised.

**Fig 8:**
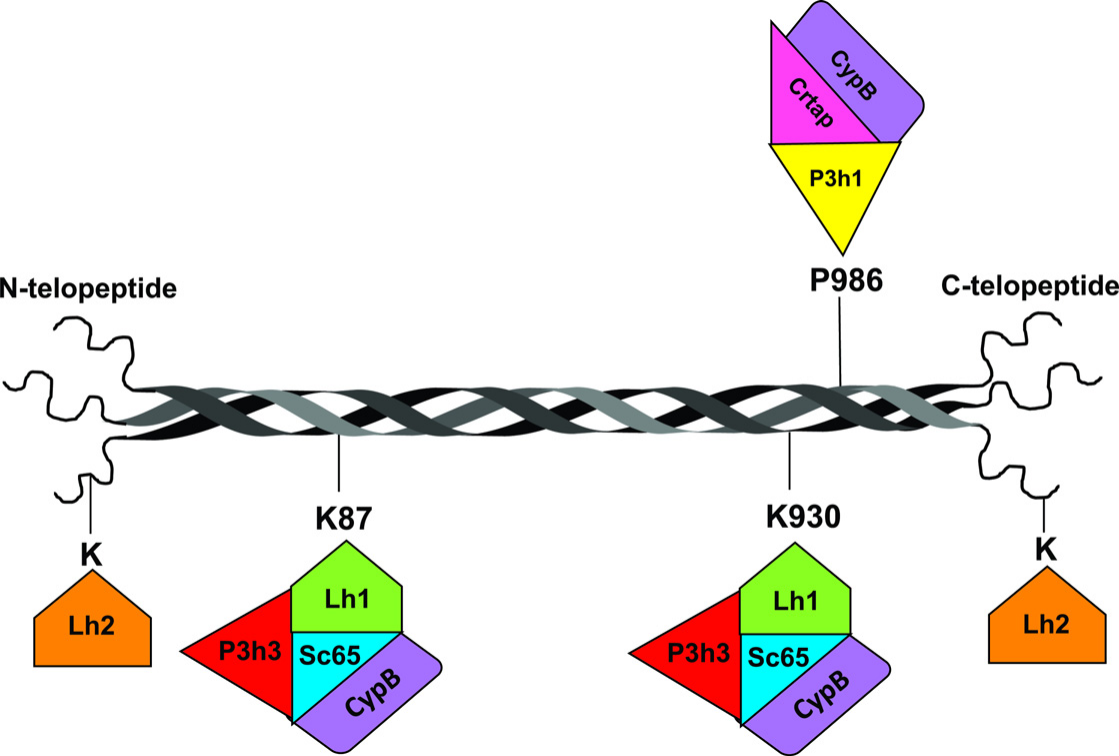
Schematic representation of a fibrillar collagen molecule in the ER. The long uninterrupted triple-helical domain is shown here already folded and without N- and C-propeptides. Depicted in different colors are some of the prolyl 3-hydroxylase enzymes (P3Hs), lysyl hydroxylase enzymes (LHs) and cyclophilin B (purple) with some of their known substrate residues. Our previous work identified the prolyl 3-hydroxylation complex that modifies P986; our current work suggests the existence of a new complex, possibly including CYPB, responsible for the hydroxylation of K87 and K930. Evidence indicates that CRTAP and SC65 act as unique orchestrators of essential molecular complexes for collagen post-translational modification. (LH2 is likely to also act within a protein complex but direct interactions have not yet been published).

Given the current evidence for a distinct complex between SC65 and P3H3 that involves LH1, along with our previous characterization of the prolyl 3-hydroxylation complex (CRTAP/P3H1/CYPB) [17], it is becoming clear that multiple high molecular weight complexes involved in regulating collagen post-translational modification have evolved in the ER (Fig 8). It is interesting to note that among the known and suspected proteins involved in these 2 complexes, only P3H1 and P3H3 have ER retention signal peptides (KDEL or REEL, respectively). Complex formation therefore may have the added function of helping retain the other proteins in the ER.

It remains to be seen, given that three prolyl 3-hydroxylases and three lysyl hydroxylases are expressed differentially in vertebrate tissues [32,33,34], whether other combinations of protein subunits are possible. In terms of clinical significance, loss of CRTAP causes a pronounced skeletal phenotype in mice and severe osteogenesis imperfecta in humans [17,35]. Loss of SC65 in mice causes no obvious phenotype during embryonic development or growth; while adult *Sc65KO* mice showed significant osteopenia [19], the most pronounced defect was in skin, consistent with the underlying pathobiochemistry revealed in skin collagen. The under-hydroxylation of lysines at triple-helical domain cross-linking sites, and resulting inversed ratio of HP/LP cross-links in bone collagen closely resembles the collagen pathology in Ehlers-Danlos Syndrome type VI (EDS VI) caused by *PLOD1* (encodes LH1) mutations (for a review see [36]). In the latter disorder, telopeptide domain lysine hydroxylation is not affected, only triple-helical domain [21,37]. The clinical phenotype includes soft skin, lax joints and kyphoscoliosis [38]. It is possible, therefore, that mutations in *SC65* (and *LEPREL2*) may be responsible for mild EDS variants sharing features of EDS VI but without a *PLOD1* mutation.

A possible mechanism that might operate to affect collagen cross-linking and hence tissue material properties selectively in skin would be through the lack of glycosylation of the K87 lysines in α1(I) and α2(I) chains. We interpret the increase in γ_112_ trimer and decrease in β_12_ dimer (Fig 2) as a consequence of altered cross-link placement in fibrils. Normally, skin type I collagen has a fully glycosylated α1(I) K87 which forms an aldimine cross-link with an α1(I)C-telopeptide allysine in fibrils after lysyl oxidase action. In *Sc65KO* skin, α1(I) and α2(I) K87 have no sugars attached and are only partially hydroxylated (Fig 3). This potentially could enable two α1(I)C-telopeptides in the same molecule to interact and form an intramolecular aldol (but not when α1(I)K87 of neighboring molecules are glycosylated). Such a shift in bond placement would result in the enrichment of native molecules extracted by dilute acetic acid that have intramolecular aldols at both ends which would run as a γ_112_ trimer on SDS-PAGE.

In summary, our studies of SC65 have revealed its importance in fibrillar collagen processing. There is clearly still much to learn about the complexity of protein interactions that have evolved to regulate the diversity in collagen post-translational quality and function evident between different tissue types.

## Methods

### Sc65 KO mice generation and genotyping

Due to the close proximity of the *Fkbp10* gene to the transcription start site of *Sc65*, the *Sc65* conditional targeting construct (using the vector p-flrt-neo-2XloxP) was designed to introduce loxP sites flanking the last 2 exons (7 and 8) of *Sc65*. This strategy eliminates the poly-A signal and creates an unstable transcript upon Cre-recombinase activity, and was successfully used at the University of Connecticut Health Center Gene Targeting and Transgenic Facility (Dr. Siu-Pok Yee, Director) where the construct was electroporated into mouse ES cells derived from F1(C57B6/jx129sv) embryos. Drug-resistant clones were screened for homologous recombination by long-range PCR using primer sets on both the 5' and 3' end of the gene. Confirmed positive clones were expanded and used to generate chimeric animals by ES-morula (obtained from CD1) aggregation. Chimeric males derived from two independent clones (1E6 and 2G4) were bred with Rosa26-Flpe females (Jax stock no. 003946 that had been backcrossed with C57BL6/j for over 20 generations) for germline transmission and removal of the Neo cassette to generate the final *Sc65* floxed allele. To generate a global *Sc65* inactivation, males carrying the floxed allele were then mated with Hprt-Cre females (Jax stock no: 004302, which has been backcrossed for over 20 generations with C57BL6/j). Pups from these females were heterozygous for the KO allele, and were bred as needed to generate heterozygote, homozygote mutant or littermate control wild type mice. The excision into the *Sc65* locus was verified by PCR analysis using long-range PCR. PCR genotyping was done using the GoTaq PCR kit and reagents (Promega, Madison, WI, USA) and a Master cycler PCR machine to run the samples (Eppendorf AG, Hamburg, Germany). The use of laboratory mice was approved by the University of Arkansas for Medical Sciences (UAMS) IACUC committee. Mice were housed in a pathogen free facility with 12h light/dark cycle with unlimited access to water and standard chow diet.

### Cell lines, expression constructs and quantitative PCR

714 mouse embryonic fibroblasts [20] and primary murine skin fibroblasts cells were grown in DMEM; primary cells from murine calvariae were grown in α‐MEM. All media contained 4500mg/L glucose and 110mg/mL sodium pyruvate (HyClone, Thermo Scientific, Pittsburgh, PA, USA) and were supplemented with 10% fetal bovine serum (FBS), L‐glutamine (2mM), 100units/mL penicillin, and 100mg/mL streptomycin. Primary osteoblasts from calvaria from 2–5 day-old pups were obtained using standard procedures as previously described [17]. Primary murine fibroblasts were isolated from 2–5 day old pups from ear snips that were minced in 0.25% trypsin and allowed to digest for 1 hour at 37°C, plated in 6 well dishes and allowed to expand. Primary fibroblasts were also treated (where indicated) with cycloheximide at a concentration of 100ug/ml and cells were collected 12hr after treatment. RNA was extracted using TriPure Isolation reagent according to the manufacture’s (Sigma Aldrich) protocol. For Real Time PCR, cDNA was synthesized from 1ug of RNA using the First Strand cDNA Synthesis Kit (Roche). cDNA samples were diluted 1:5 and quantitative PCR was performed with the LightCycler version 1.5 instrument using Roche Applied Science reagents according to manufacturer recommendations. Primer sequences were as follows: for SC65—FWD: 5’-ATGCAGCAGAACCTGGTATATT-3’ and RVS: 5’-GTCTGGTTGTGGTAGAGCATA-3’; for GAPDH—FWD: 5’-GCAAGAGAGGCCCTATCCCAA-3’ and RVS: 5’-CTCCCTAGGCCCCTCCTGTTATT-3’. The pCMV-SC65-DDK-C plasmid (murine, SC65-FLAG) was purchased from TransOMIC Technologies, Huntsville, AL, USA). Full-length LH1 and CypB cDNAs and exons 1–6 of *Sc65* were amplified from 714 mouse embryonic fibroblasts and cloned into the pCMV-HA-C plasmid (TransOMIC Technologies, Huntsville, AL, USA) using the In-Fusion HD Cloning kit (Clontech, Mountain View, CA, USA) according to the manufacturer’s protocol and sequence verified.

### Co-immunoprecipitation assays

#### Immunoprecipitation of Sc65-flag and interacting partners for proteomic mass spectrometric analysis

714 mouse embryonic fibroblasts cells were plated in 10cm dishes (5x10^6^) and allowed to adhere overnight. Cells at 70% confluency were transfected with SC65-Flag vector using Xtreme Gene (Roche, Indianapolis, IN, USA) according to the manufacturer’s protocol. Transfection was performed using 10μg of DNA with 10μl of Xtreme gene transfection reagent diluted in serum free medium. Cells were harvested 24 hours after transfection. Plates were washed once in sterile PBS and trypsinized. Following centrifugation at 1,000xg, pellets were rinsed with PBS and resuspended in lysis buffer (1 mM EDTA, 0.5% Triton X-100, PBS). Cell lysates were incubated on ice for 10 minutes and subjected to sonication. Anti-Flag antibody (cat# 635691, Clontech) was conjugated to Epoxy Dynabeads (Life Technologies, Grand Island, NY, USA) for immune-precipitation of SC65-Flag. Beads were washed with 100mM sodium phosphate pH 7.4 for ten minutes on a rotator at room temperature. 30μg of anti-Flag antibody was added to 100mM sodium phosphate pH 7.4 then mixed with 3M ammonium sulfate. Solution was removed from Dynabeads and antibody mixture was added and allowed to rotate overnight at 30°C. The following day beads were washed once with 100mM glycine HCl pH 2.5 and once with 10mM Tris pH 8.8. Beads were quickly washed once with 100mM triethylamine and thoroughly washed with 0.5% Triton-X 100 /PBS. Conjugated beads were added to cell lysates and allowed to incubate on a rotator for four hours at 4°C. Following incubation, beads were removed and washed with cold lysis buffer. For elution of bound proteins, beads were incubated with 0.5N ammonium hydroxide for five minutes at room temperature. Eluted proteins were speed vacuumed overnight and resuspended in BME sample buffer. Samples were resolved by SDS–PAGE/Coomassie-staining and excised as 2mm bands from the entire gel lane and further processed for mass spectrometry.

#### Proteomic mass spectrometry

Gel pieces excised from the Coomassie blue gel were de-stained in 50% methanol, 100mM ammonium bicarbonate, followed by reduction in 10mM Tris(2-carboxyethyl) phosphine and alkylation in 50mM iodoacetamide. Gel slices were then dehydrated in acetonitrile, followed by addition of 100ng porcine trypsin (Promega) in 100mM ammonium bicarbonate and incubation at 37°C for ~14 hours. Peptide products were then acidified in 0.1% formic acid. Tryptic peptides were analyzed by nanoflow LC-MS/MS with a Thermo Orbitrap Velos mass spectrometer equipped with a Waters nanoACQUITY LC system as described previously [39]. Mascot results were uploaded into Scaffold 4 (version 4.00.01) for viewing the proteins and peptide information. A false discovery rate of 1% was used as the cut off value. To measure enrichment of a protein, we used spectral counting normalized by the Normalized Spectral Abundance Factor (NSAF) method described previously by Byrum S. et al [39]. Following log transformation of NSAF values, fold change values were generated by dividing a protein’s enrichment value in the immune-precipitated sample by its value in our negative control sample. Proteins with a fold change >1.5 were considered significantly enriched.

### Collagen extraction, SDS-PAGE, crosslink and mass spectral analyses

#### Tissue preparation

Bone and skin were scraped clean and defatted with chloroform/methanol (3:1 v/v), bone was demineralized in 0.1M HCl, all steps at 4°C. Collagen was solubilized from skin by 3% acetic acid extraction at 4°C and subsequent heat denaturation at 90°C in SDS-PAGE sample buffer. Bone collagen was solubilized directly by heat denaturation in SDS-PAGE sample buffer. Demineralized bone and skin were also digested with bacterial collagenase as described [40] with the addition of 10% acetonitrile to the digestion buffer, and collagenase generated peptides were separated by reversed-phase HPLC (C8, Brownlee Aquapore RP-300, 4.6mm x 25cm) with a linear gradient of acetonitrile:n-propanol (3:1 v/v) in aqueous 0.1% (v/v) trifluoroacetic acid [41].

#### SDS-PAGE

The method of Laemmli [42] was used with 6% gels for extracts of tissue collagen. Type I α-chains from skin and bone extracts were cut from gels and digested with trypsin in-gel for mass spectral analyses.

#### Cross-link analysis

Bone was hydrolyzed in 6N HCl, dried, dissolved in 1% (v/v) n-heptafluorobutyric acid and analyzed by C18 reverse-phase HPLC as described [43].

#### Analytical mass spectrometry

Electrospray LC-MS/MS was performed on tryptic peptides and individual collagenase HPLC fractions using an LTQ XL ion-trap mass spectrometer (Thermo Scientific) equipped with in-line liquid chromatography using a C4 5um capillary column (300um x 150mm; Higgins Analytical RS-15M3-W045) eluted at 4.5ul min. The LC mobile phase consisted of buffer A (0.1% formic acid in MilliQ water) and buffer B (0.1% formic acid in 3:1 acetonitrile:n-propanol v/v). The LC sample stream was introduced into the mass spectrometer by electrospray ionization (ESI) with a spray voltage of 4kV. Proteome Discoverer search software (Thermo Scientific) was used for peptide identification using the NCBI protein database. Proline and lysine modifications were examined manually by scrolling or averaging the full scan over several minutes so that all of the post-translational variations of a given peptide appeared together in the full scan.

### Direct co-immunoprecipitation assays

To confirm candidate interactors of SC65, 714 cell lines stably expressing SC65-Flag were generated as follows. The SC65-DDK-C sequence (from the pCMV-Sc65-DDK plasmid) was sub-cloned into the pLEN expression vector [44] (carrying G418 resistance) utilizing the In-fusion HD Cloning kit as above. The construct was linearized by digestion with Nde1 (New England Biolabs, Ipswich, MA, USA), transfected into 714 cells with Lipofectamine 3000 (Life Technologies) according to the manufacturer’s protocol and maintained in G418 selection (Sigma Aldrich, St. Louis, MO, USA). A negative control cell line was generated by transfecting an empty pLen vector (EV) into 714 cells. For direct co-immunoprecipitation of LH1-HA, five 10cm dishes of both cell lines were transfected with 20ug of LH1-HA with Lipofectamine 3000 (Life Technologies) according to the manufacturer’s protocol. Forty-eight hours after transfection, immune-precipitation of SC65-FLAG or LH1-HA was performed using the Thermo Scientific Magnetic Crosslinking IP Kit with modifications. Cells were lysed in IP Lysis/Wash buffer to a volume of 2ml, centrifuged for 10min @ 10,000 RPM at 4°C. The supernatant was incubated with 10-15ug of anti-DDDK (Bethyl Laboratories, Montgomery, TX, USA) or anti-HA (Santa Cruz Biotechnologies, Dallas, TX, USA) antibody overnight at 4°C. A/G magnetic beads were added to the lysate/antibody mixture and allowed to conjugate for 4 hours at 4°C. Beads were washed twice with IP/Lysis wash buffer and ultrapure water. Beads were incubated in an elution buffer (pH 2) for 10min and elutions were run on 10% SDS-PAGE gels and immunoblotted for target proteins. Co-immunoprecipitation of P3H3 and CYPB-HA was carried out the same as LH1-HA except endogenous P3H3 was immunoprecipitated utilizing a polyclonal P3H3 antibody (2.5ug, ProteinTech, Chicago, IL, USA). To determine if LH1 and P3H3 interact, 714-pLen- EV cells were transfected with LH1-HA as above in the presence of ascorbic acid and lysed after 48hours. Immunoprecipitation was performed as above using anti-HA and P3H3 antibodies. Controls for these experiments included untransfected 714-pLen-EV cells incubated with HA antibody to determine non-specific binding proteins and LH1-HA transfected cells that were incubated with beads alone to determine any proteins non-specifically binding to the beads.

### Size exclusion chromatography

To further pursue the nature of the putative SC65/P3H3/LH1/CYPB complex we performed a preparative, high resolution size exclusion chromatography. For this experiment, five to ten 100mm cell culture dishes of confluent 714 cells stably transfected with SC65-Flag and treated for 72 hours with ascorbic acid (100μg/ml) were used in multiple large IP experiments using a monoclonal antibody against Flag (see “Co-immunoprecipitation assays” in Methods), (Bethyl). To maintain its integrity, the protein complex pulled down in the IP was eluted from the magnetic beads by competitive binding with an excess of Flag peptide (500ug/ml, Sigma Aldrich). Size exclusion chromatography was performed using a Superdex 200 Increase 10/300 GL column (approximate bed volume of 24ml) with the AKTA purifier FPLC (GE Healthcare). All experiments (standards and samples) were performed at room temperature following the manufacture’s protocol. The column was first equilibrated with 2 column volumes (CV) of room tempered water followed by equilibration with 2 CV of eluent utilized in each experiment (50mM phosphate buffer containing 0.15M NaCl pH7.4). In order to standardize the column, a Gel Filtration Calibration Kit (GE Healthcare) containing Thyroglobulin, Ferritin, Aldolase, Conalbumin and Ovalbumin was used. The void volume (Vo) at ~8.5 mL was used with the elution volume (Ve) to generate a protein standard curve to calculate the size of eluted proteins (y = -0.3048x+0.9212, R^2^ = 0.9937). The eluted suspension from the immunoprecipitation was then loaded onto the column and ran at the recommended flow rate of 500ul/min. Fractions of 500ul each were eluted from the column, further concentrated by Trichloroacetic Acid (TCA, Sigma Aldrich) precipitation, re-suspended in PBS, run on a SDS-Page and blotted with relevant antibodies (see above “Western blotting”).

### Western blotting

Tissues and primary cell cultures were lysed into RIPA buffer (50mM Tris‐HCl pH 7.5, 150mM NaCl, 0.1% SDS, 1mM EDTA, 0.5% sodium deoxycholate, and 1% Triton X‐100) containing a cocktail of protease inhibitors including EDTA (cAMRESCO, Solon, OH, USA). Lysates were centrifuged (15,000g) and supernatants collected and quantified using the Bio‐Rad protein assay dye reagent (Bio‐Rad, Hercules, CA, USA). Proteins were separated by 10% SDS-PAGE gels according to standard techniques, transferred to a nitrocellulose membrane and blocked for 30min in 5% milk. Primary antibodies were Sc65 (cat# 15288-1-AP, ProteinTech), β-actin (cat# A00702, GenScript, Piscataway, NJ, USA), LH1 (cat#NBP2-38770, Novus Biologicals, Littleton, CO, USA), P3H3 (cat# 16023-1-AP, ProteinTech), anti-DDDK (cat# A190-102A, Bethyl Laboratories), anti-HA (cat# sc-7392, Santa Cruz Biotechnologies). Secondary antibodies were goat anti‐rabbit or anti‐mouse IRDye 680LT (LICOR Biosciences, Lincoln, NE, USA). Membranes were scanned using a LICOR Odyssey instrument.

### Histology, immunohistochemistry and immunofluorescence

Femur, tibiae and skin were harvested and fixed in 10% buffered formaldehyde. After demineralization in 30% EDTA or non-decalcified femur or tibiae, the specimens were dehydrated, cleared and embedded in either paraffin or in Methyl methacrylate according to standard procedures [45]. Paraffin embedded sections were cut at 5 microns on a Leitz1512 microtome and specimens embedded in Methyl Methacrylate were cut on a Leica RM2255 automatic heavy duty retractable microtome using a D-profile tungsten carbide knife. Sections were mounted on Silane+ slides (Newcomer’s Supply, Middleton, WI) using Haupt’s media to allow the sections to adhere better to the slides. Skin was stained with hematoxylin and eosin and both bone and skin sections were stained with Sirius red. A 0.1% solution of Sirius red (Direct Red 80, Sigma-Aldrich) was prepared in saturated aqueous solution of picric acid. After staining, sections were rinsed in acidified water (87.5mM acetic acid) and dehydrated in absolute alcohol, cleared and mounted in synthetic resin (DPX Mountant for histology, Sigma-Aldrich). Sirius red stained sections were analyzed under polarized light and images were taken using a Zeiss AxioImager equipped with DIC filter, polarizer, Zeiss AxioCam MRc5 digital camera and Zeiss Axiovision software.

For immunohistochemistry staining, bone and skin sections mounted on slides were placed in Citrate Buffer pH 6.0 which had been heated to boiling in a microwave oven and allowed to cool for 30 minutes at room temperature. Endogenous peroxidase was blocked using the Peroxidase Solution supplied in the CTS008 Anti-Goat HRP-DAB Cell and Tissue Staining Kit (R&D Systems). After 5’ the slides were washed with PBS pH7.4, blocked with the blocking buffer supplied in the kit and incubated overnight with the antibody. The next morning the sections were washed in PBS pH7.4, secondary antibody applied incubated for 30 minutes, washed and DAB applied. After development of DAB the sections were washed with distilled water, counterstained with Mayer’s Hematoxylin, dehydrated, cleared and mounted with Permaslip. Pictures were taken using a Zeiss AxioImager equipped with Zeiss AxioCam MRc5 digital camera and Zeiss Axiovision software.

For immunofluorescence staining, skin sections were de-paraffinized and hydrated in a series of xylene and ethanol incubations according to standard protocols. Sections were heated in 10mM citrate buffer (pH 6.0) until boiling and let incubate for 30 minutes until cooled. Sections were then blocked in normal serum blocking buffer (3% goat serum, 1mg/ml BSA, 0.1% Triton-X) for 1 hour. Sections were then incubated with SC65 primary antibody (ProteinTech) overnight. After washing, sections were incubated with AlexaFluor 488 goat anti-rabbit secondary antibody (Life Technologies) for 30 minutes followed by washing and mounting in DAPI Fluoromount (SouthernBiotech, Birmingham, AL, USA). Images were acquired on a Zeiss AxioImager scope.

### Micro-CT analyses

All μCT analyses were consistent with current guidelines for the assessment of bone microstructure in rodents using micro-computed tomography [46]. Formalin-fixed tibiae and femurs from WT and *Sc65KO* mice 6 months old (n = 9–10) were imaged using a Micro-CT 40 (Scanco Medical AG, Bassersdorf, Switzerland) using a 12μm isotropic voxel size in all dimensions. The region of interest selected for analysis comprised 240 transverse CT slices representing the entire medullary volume extending 1.24mm distal to the end of the primary spongiosa with a border lying 100μm from the cortex. Three-dimensional reconstructions were created by stacking the regions of interest from each two-dimensional slice and then applying a gray-scale threshold and Gaussian noise filter (sigma 0.8, support 1, threshold 245) as described [45] using a consistent and pre-determined threshold with all data acquired at 55kVp, 114mA, and 200ms integration time. Fractional bone volume (bone volume/tissue volume; BV/TV) and architectural properties of trabecular bone such as trabecular thickness (Tb.Th, μm), trabecular number (Tb.N, mm^-1^), and connectivity density (Conn. D, 1/mm^3^) were calculated using previously published methods [45]. Femoral and tibial cortical geometry was assessed in a 1mm-long region centered at the femoral midshaft. The outer contour of the bone was found automatically using the built-in manufacturer’s contouring tool. Total area was calculated by counting all voxels within the contour, bone area by counting all voxels that were segmented as bone, and marrow area was calculated as total area minus bone area. This calculation was performed on all 25 slices (1 slice = 12.5μm), using the average for the final calculation. The outer and inner perimeter of the cortical midshaft was determined by a three-dimensional triangulation of the bone surface (BS) of the 25 slices, and cortical thickness and other cortical parameters were determined as described.

### Electron microscopy analyses

Dorsal skin samples were collected from 7 months old WT and *Sc65KO* animals (n = 4) and fixed overnight at 4°C in 2.5% glutaraldehyde (Electron Microscopy Sciences (EMS), Hatfield, PA, USA), 0.05% malachite green (Sigma Aldrich) in 0.1M sodium cacodylate buffer, pH 7.2 (EMS). After washing with 0.1M sodium cacodylate buffer, the samples were post-fixed for 2 hrs with 1% osmium tetroxide (EMS), 0.8% potassium hexaferrocyanide (Sigma Aldrich) for 2 hours and 1% tannic acid (EMS) for 20 min. The samples were rinsed with molecular grade water and stained with 0.5% uranyl acetate (EMS) for 1 hour then dehydrated with a graded alcohol series and propylene oxide before embedding in Araldite/Embed 812 (EMS). Sections (50nm) were cut on a Leica UC7 ultra-microtome and collected on formvar carbon coated slot grids and post-stained with uranyl acetate and lead citrate. Imaging was done using a Technai F20 (FEI, Netherlands) at 80kV. Analyses of collagen fibril diameter (>200 fibrils/mouse) and of collagen inter-fibril space were performed with the Leica Application Suite v 3.0 image analysis software (Leica Microsystems, Milan, Italy) on 5 sections for each mouse (n = 3) at the magnification 19000X.

### Skin tension test

Samples were prepared from the dorsal skin of adult (7 month-old) males and females *Sc65KO* and WT mice (n = 7 or more, see Fig 7). The skin was harvested and cut into about 2cm wide by 4cm long pieces. The long axis of the sample coincided with the cranio-caudal axis of the mouse. The samples were clamped between two custom built aluminum fixtures at the superior and inferior ends (see Fig 7G). Tension tests were performed on a MTS 858 Bionix Test System (Eden Prairie, MN) servo-hydraulic material test machine. The samples were preloaded to 0.2Newtons and then loaded to failure in tension at a constant rate of 10mm/min. Peak load and stiffness (calculated as the slope of the load/deflection curve) were recorded using TestWorks 4 software (Eden Prairie, MN).

### Statistical analysis

All parameters measured are presented as mean ± Standard Deviation and were analyzed with the Student’s t-test using a two-tailed distribution and two-sample equal variance as appropriate. All calculations were performed utilizing Microsoft Excel. P values <0.05 were considered statistically significant and reported as such.

### Ethics statement

All animal work (i.e. on rodents) performed in this study was conducted in accordance to local, State and US Federal regulations. The UAMS IACUC has approved the animal protocol (AUP#3349 entitled "Role of the Leprecan Genes in Skeletal Formation") describing all the procedures performed in this study. Mice were euthanized to harvest relevant tissues according to the recommendations of the Guide for Care and Use of Laboratory Animals (8^th^ Edition).

## Supporting Information

S1 Fig Real-time PCR and western blot confirm rapid degradation of Sc65 truncated transcripts and lack of putative truncated protein products. a) cDNA prepared from WT or *Sc65KO* primary fibroblasts (N = 3–4) was analyzed by qPCR to evaluate *Sc65* expression levels. Null fibroblasts showed significantly reduced transcript levels (normalized to *Gapdh*) compared to WT. Upon treatment with cycloheximide to inhibit NMD, *Sc65* transcript levels increased to 60–65% of WT levels in Sc65KO cells. These results confirm that targeted deletion of the last two exons of *Sc65* results in quick degradation of the Sc65 transcript and a null allele. b) To exclude the potential generation of SC65 truncated protein products that could escape detection from the used polyclonal antibody, the first 6 exons of *Sc65* were cloned into a C-terminal HA tag expression vector and transfected into 714 cells. The SC65 polyclonal antibody (left panel) was indeed able to detect both endogenous SC65 as well as the truncated form.
(TIF)

S2 Fig Mass-spectrometry identification of Sc65 interactors. a) Coomassie blue gel showing separation of immune-precipitates obtained using the indicated experimental conditions. Identification and quantification of proteins in gel lanes #1 and 3 was performed by mass-spectrometry. The protein enrichment in lane #3 compared to lane #1 is represented as fold change in b; 253 proteins with fold change >1.5 were identified as candidate Sc65 interactors. In lane #3, Sc65 was 35 fold enriched, indicating the success of the IP procedure.
(TIF)

S3 Fig Size exclusion chromatography. A large scale immunoprecipitation of SC65-FLAG was run on a Superdex 200 Increase (10/300) gl column and 0.5ml fractions collected, TCA concentrated, ran on a SDS-PAGE and blotted with relevant antibodies. SC65 (detected by the Flag antibody) and P3H3 proteins were present in fractions 24–26 (equivalent to an estimated MW of about 250kDa) and in significant smaller amounts in fractions 23 and then 27–28. LH1 protein was present in fractions 28–30 with significant smaller amounts in fraction 25–27. CYPB was not detected in the fractions shown here.
(TIF)

S4 Fig Immuno-fluorescence on skin sections. Sc65 is expressed in WT dermal fibroblasts; its expression is lost in skin sections from *Sc65KO* mice.
(TIF)

S1 Table Summary of 3Hyp occupancy in type I collagens from Sc65 mouse tissues. Percentage of 3Hyp at each major substrate site in type I collagen from bone and skin. The percentages were determined based on the ratio the m/z peaks of each post-translational variant as previously described.
(DOCX)
