# Supplementary figures and images for: Sc65-Null Mice Provide Evidence for a Novel Endoplasmic Reticulum Complex Regulating Collagen Lysyl Hydroxylation
Source: PLoS Genet. 2016 Apr 27;12(4):e1006002. doi: 10.1371/journal.pgen.1006002 (PMC4847768; doi:10.1371/journal.pgen.1006002)

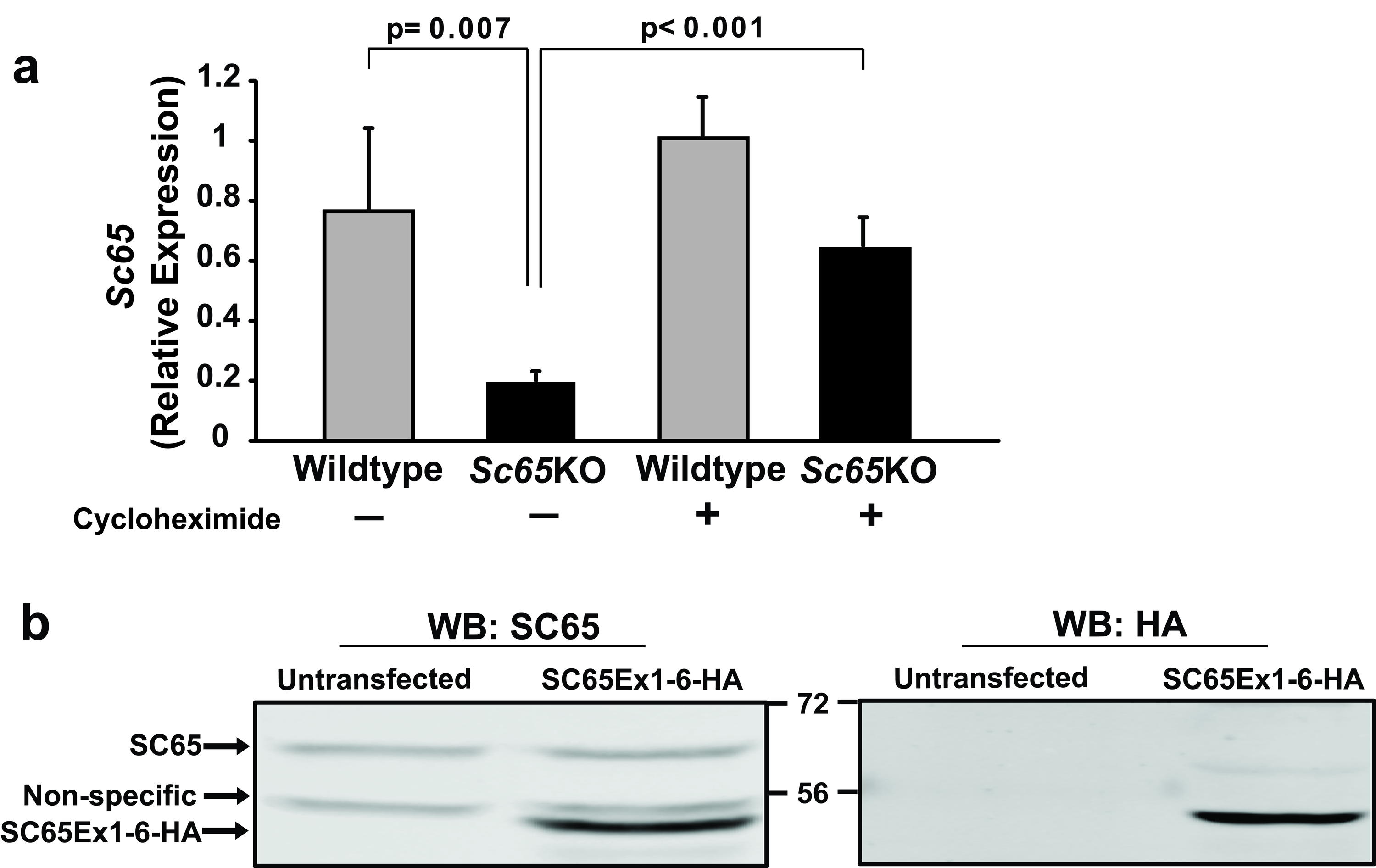

Supplement: S1 Fig — a) cDNA prepared from WT or Sc65KO primary fibroblasts (N = 3–4) was analyzed by qPCR to evaluate Sc65 expression levels. Null fibroblasts showed significantly reduced transcript levels (normalized to Gapdh) compared to WT. Upon treatment with cycloheximide to inhibit NMD, Sc65 transcript levels increased to 60–65% of WT levels in Sc65KO cells. These results confirm that targeted deletion of the last two exons of Sc65 results in quick degradation of the Sc65 transcript and a null allele. b) To exclude the potential generation of SC65 truncated protein products that could escape detection from the used polyclonal antibody, the first 6 exons of Sc65 were cloned into a C-terminal HA tag expression vector and transfected into 714 cells. The SC65 polyclonal antibody (left panel) was indeed able to detect both endogenous SC65 as well as the truncated form. (TIF) [file pgen.1006002.s001.tif]

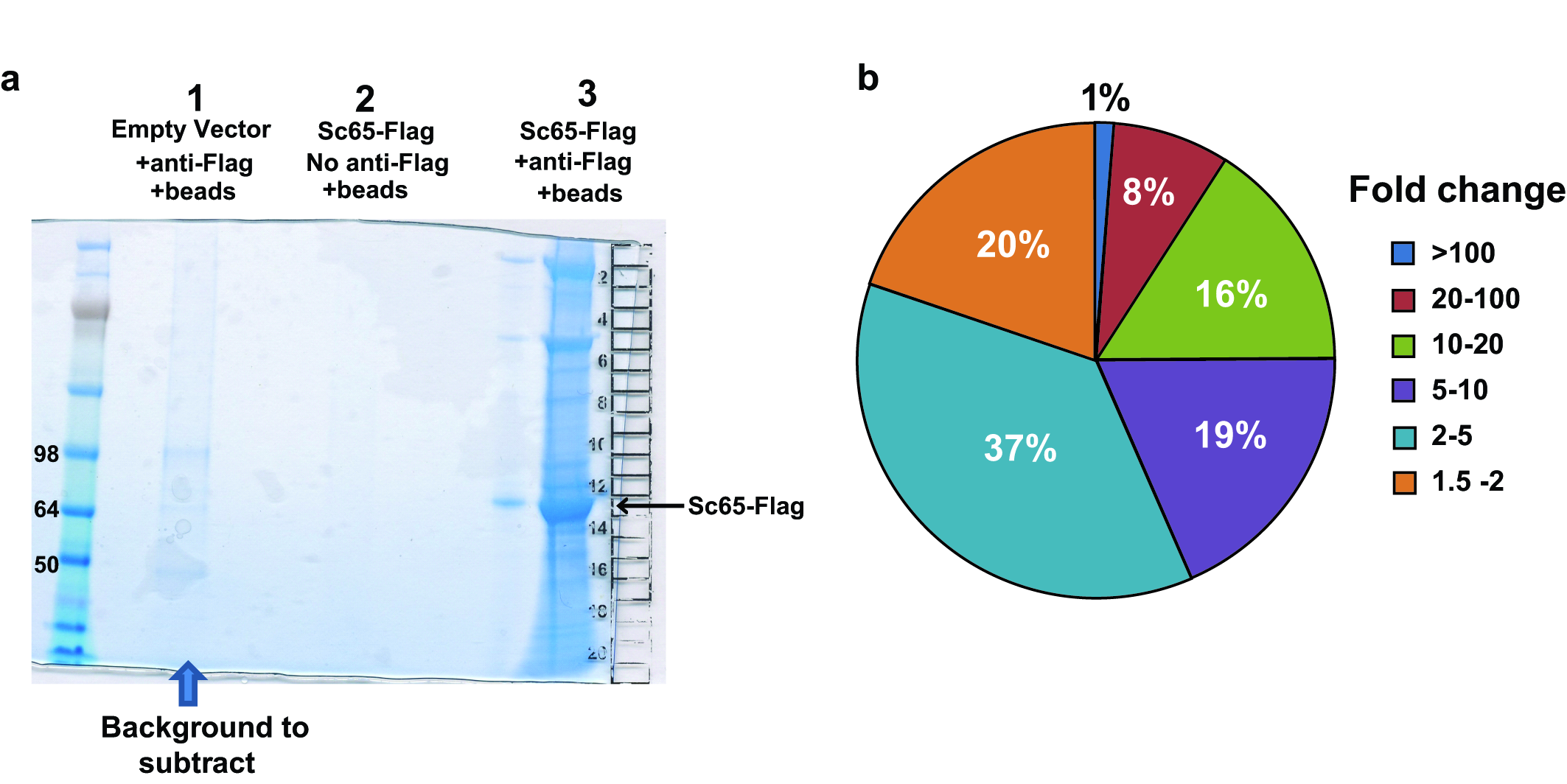

Supplement: S2 Fig — a) Coomassie blue gel showing separation of immune-precipitates obtained using the indicated experimental conditions. Identification and quantification of proteins in gel lanes #1 and 3 was performed by mass-spectrometry. The protein enrichment in lane #3 compared to lane #1 is represented as fold change in b; 253 proteins with fold change >1.5 were identified as candidate Sc65 interactors. In lane #3, Sc65 was 35 fold enriched, indicating the success of the IP procedure. (TIF) [file pgen.1006002.s002.tif]

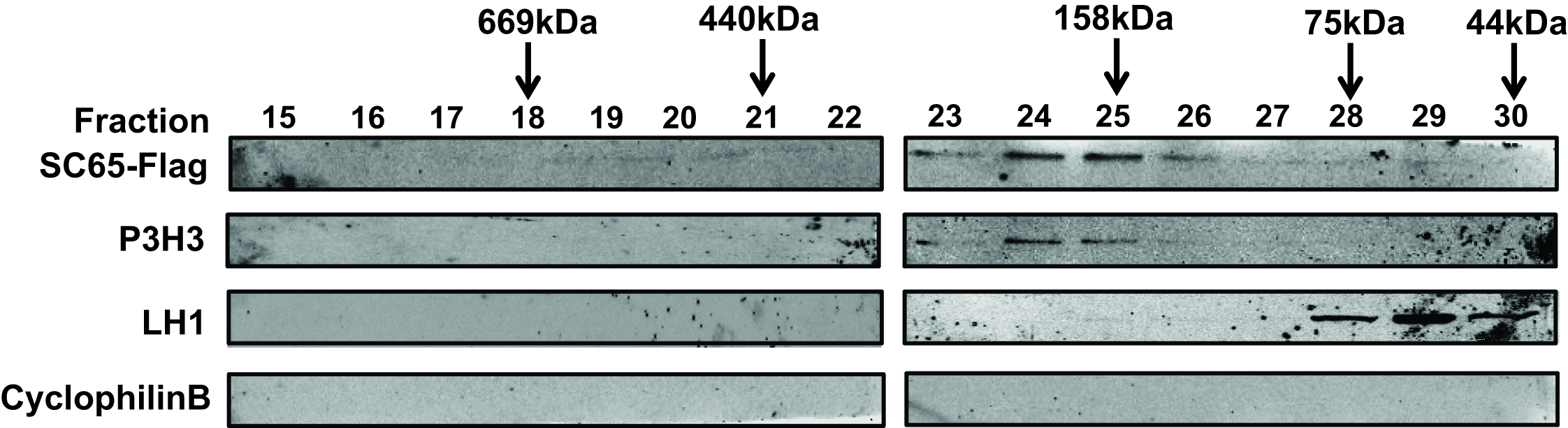

Supplement: S3 Fig — A large scale immunoprecipitation of SC65-FLAG was run on a Superdex 200 Increase (10/300) gl column and 0.5ml fractions collected, TCA concentrated, ran on a SDS-PAGE and blotted with relevant antibodies. SC65 (detected by the Flag antibody) and P3H3 proteins were present in fractions 24–26 (equivalent to an estimated MW of about 250kDa) and in significant smaller amounts in fractions 23 and then 27–28. LH1 protein was present in fractions 28–30 with significant smaller amounts in fraction 25–27. CYPB was not detected in the fractions shown here. (TIF) [file pgen.1006002.s003.tif]

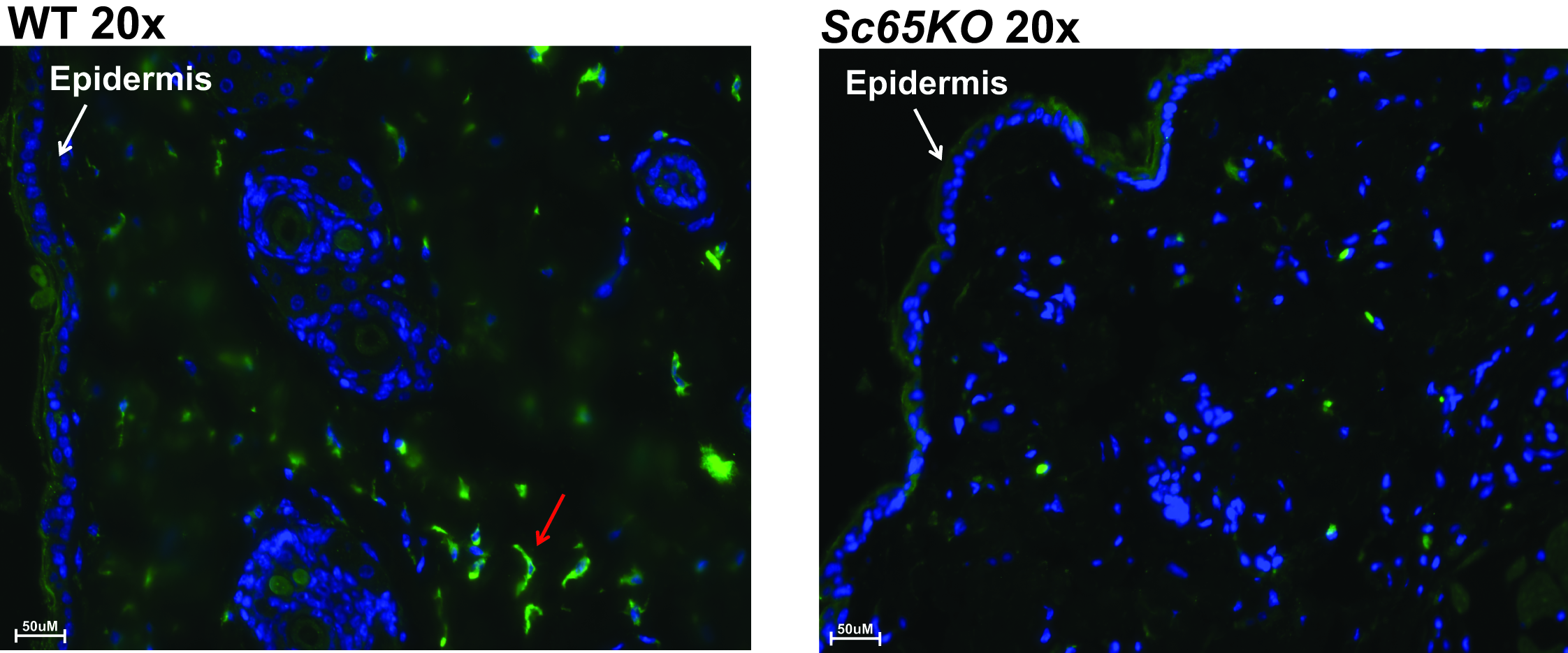

Supplement: S4 Fig — Sc65 is expressed in WT dermal fibroblasts; its expression is lost in skin sections from Sc65KO mice. (TIF) [file pgen.1006002.s004.tif]
