# Supplementary material for: Sc65-Null Mice Provide Evidence for a Novel Endoplasmic Reticulum Complex Regulating Collagen Lysyl Hydroxylation
Source: PLoS Genet. 2016 Apr 27;12(4):e1006002. doi: 10.1371/journal.pgen.1006002 (PMC4847768; doi:10.1371/journal.pgen.1006002)
Supplement: S1 Table — Percentage of 3Hyp at each major substrate site in type I collagen from bone and skin. The percentages were determined based on the ratio the m/z peaks of each post-translational variant as previously described. (DOCX) [file pgen.1006002.s005.docx]

|  | α1(I)986 | | α2(I)707 | |
| --- | --- | --- | --- | --- |
|  | Bone | Skin | Bone | Skin |
| *Sc65KO* | 99% | 90% | 25% | 5% |
| *WT* | 99% | 90% | 25% | 10% |
